# Rhino-orbital mucormycosis due to *Apophysomyces ossiformis* in a patient with diabetes mellitus: a case report

**DOI:** 10.1186/s12879-020-05337-4

**Published:** 2020-08-18

**Authors:** Erick Martínez-Herrera, María Guadalupe Frías-De-León, Angélica Julián-Castrejón, Luis Cruz-Benítez, Juan Xicohtencatl-Cortes, Rigoberto Hernández-Castro

**Affiliations:** 1 Hospital Regional de Alta Especialidad de Ixtapaluca, Estado de México, Mexico; 2 Laboratorio de Bacteriología Intestinal, Hospital Infantil de México “Dr. Federico Gómez”, 06720 Ciudad de México, Cuauhtémoc Mexico; 3 Departamento de Ecología de Agentes Patógenos, Hospital General “Dr. Manuel Gea González”, Sección XVI, Tlalpan 14080, Ciudad de México, Mexico

**Keywords:** *Apophysomyces ossiformis*, Diabetes mellitus, Invasive fungal disease, Mucormycosis

## Abstract

**Background:**

The most common aetiological agents of mucormycosis are *Rhizopus*, *Mucor*, *Apophysomyces* and *Lichtheimia*. *Apophysomyces* is comparatively rare, as it has been reported in less than 3% of mucormycosis cases. The genus *Apophysomyces* includes six species, and only *A. elegans*, *A. mexicanus*, *A. variabilis* and *A*. *ossiformis* have been reported to cause infections in both immunocompetent and immunocompromised patients.

**Case presentation:**

We present a case of a 46-year-old male patient with bilateral blepharoedema, corneal opacity in the left eye and poorly controlled diabetes mellitus. The patient was subjected to total maxillectomy, exenteration of the left orbit and treatment with liposomal amphotericin B. Direct mycological analysis with KOH 10% revealed hyaline, coenocytic, long and wide hyphae. *Apophysomyces ossiformis* was identified from maxillary biopsy using 18S-ITS1–5.8S-ITS2-28S rRNA gene amplification and sequencing. The patient requested to be transferred to another hospital to continue treatment, where he died on the ninth day after admittance.

**Conclusion:**

To the best of our knowledge, this is the first case of rhino-orbital mucormycosis due to *A. ossiformis* with a fatal outcome. This case reveals the need to identify the fungus causing mucormycosis with molecular methods to identify adequate treatment therapies for patients with this infection.

## Background

Mucormycosis is an invasive infection caused by Mucorales [1]. The main risk factors for this mycosis are diabetes mellitus type 2, immunosuppression, neutropenia, metabolic acidosis, leukaemia, lymphoma, and organ transplants [2]. The clinical presentation can be pulmonary, gastrointestinal, cutaneous, and disseminated; however, the most frequent presentation is rhino-cerebral, with a high mortality rate [1, 3]. The most common aetiological agents are *Rhizopus*, *Mucor*, *Apophysomyces* and *Lichtheimia*. *Apophysomyces* is comparatively rare, as it has been reported in less than 3% of mucormycosis cases [4]. The genus *Apophysomyces* includes six species (*A. elegans*, *A. mexicanus*, *A. ossiformis*, *A. thailandensis*, *A. trapeziformis*, and *A. variabilis*), and only *A. elegans*, *A. mexicanus*, *A*. *ossiformis* and *A. variabilis* have been reported to cause infections in both immunocompetent and immunocompromised patients [5–7]. We present the first case of rhino-orbital mucormycosis due to *A. ossiformis* in a patient with poorly controlled diabetes from the central region of Mexico.

## Case presentation

**Fig. 1:**
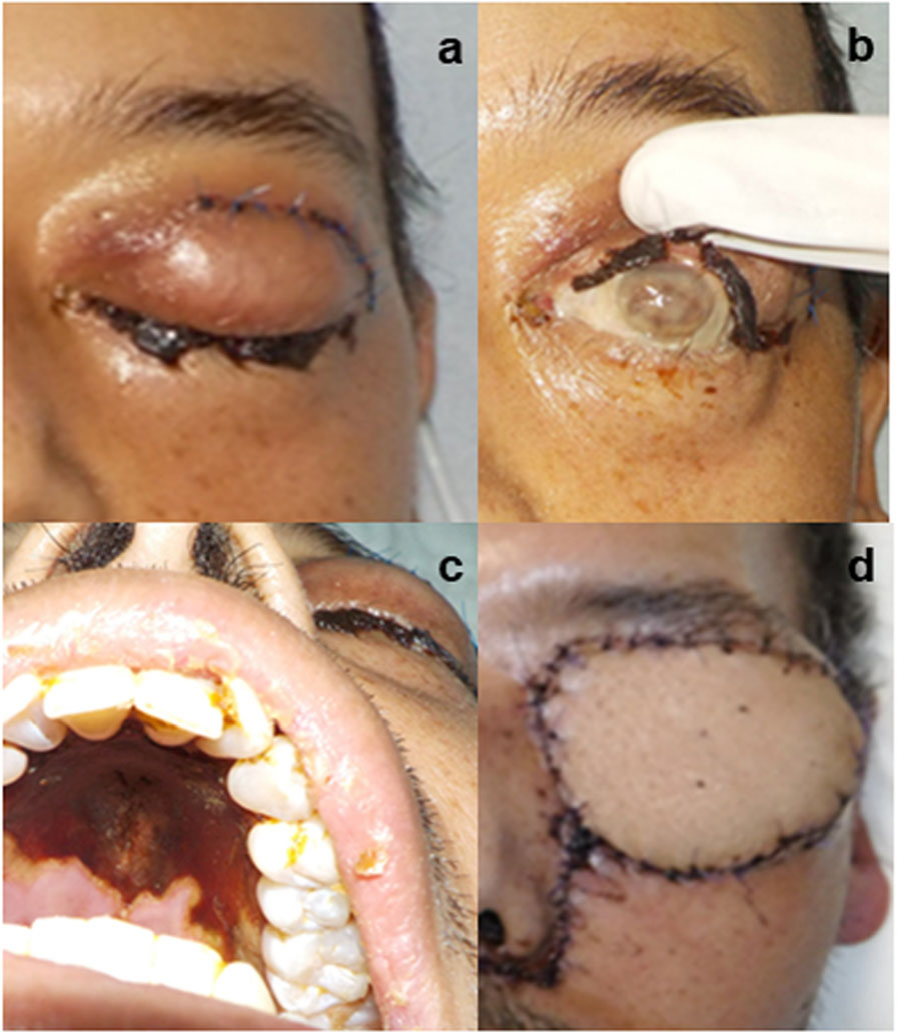
Acute inflammation of the orbit of the left eye **a**; corneal opacity of the left eye **b**; oral cavity with necrotic ulcers **c**; and total maxillectomy and exenteration of the left orbit **d**

A 46-year-old male patient presented with a conjunctival infection and probable Reis-Bücklers dystrophy (clinically characterized by superficial corneal opacities, recurrent erosions and significant visual impairment), and multiple antimicrobial treatments showed no improvements. At admittance to our institution, the patient reported a headache and moderate pain in the bilateral orbitofrontal region radiating to the occipital region. He had a history of uncontrolled diabetes mellitus type 2 for 3 years and poor treatment compliance. A physical examination revealed fever, chills, and general malaise, with moderate bilateral ocular pain, nasal congestion, dehydrated nasal mucosa, odynophagia, cough, dyspnoea, hemoptysis, bilateral blepharoedema, and corneal opacity in the left eye (Fig. 1a, b). A black eschar was seen on the palate (Fig. 1c).

The patient was evaluated in the Ophthalmology Department, where a lacrimal gland biopsy was performed, and acute inflammation of the orbit of unknown origin was observed. A facial CT scan was performed and revealed pansinusitis with deformation of the left ocular globe, luxation of the crystalline, maxillary affectation, and deformation of the floor of the bilateral orbital bone occupied by both maxillary sinuses. The recommended starting treatment was liposomal amphotericin B (5 mg/kg/day) and the initiation of glycaemic control due to probable mucormycosis. A biopsy was taken, and surgical debridement was performed with total maxillectomy and exenteration of the left orbit; a microvascularized femur graft was obtained to reconstruct the left orbit (Fig. 1d). During surgery, necrosis of the whole maxilla as well as the orbit floor, including the nasal septum, was observed. Samples of the maxillary tissue, ocular globe, and nasal septum were sent for histopathological analysis.

**Fig. 2:**
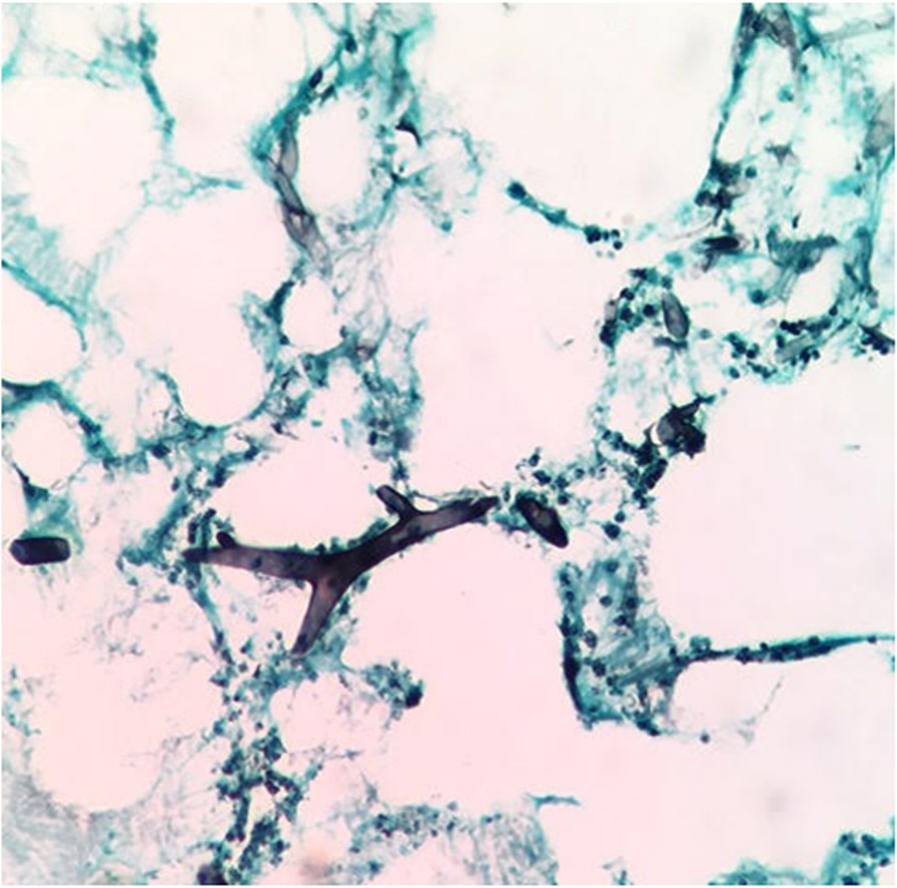
Histopathological findings: wide and long non-septate hyphae with irregular contours (Grocott-Gomori staining, × 40)

Haematoxylin-eosin and Grocott-Gomori staining allowed the observation of wide hyphae with irregular contours (Fig. 2), and a direct mycological study with KOH 10% revealed hyaline, coenocytic, long and wide filaments, compatible with members of the order Mucorales. No mycological culture was performed.

Molecular identification was performed by the amplification and sequencing of DNA fragments containing the 18S rRNA gene, the internal transcribed spacer regions (ITS1, 5.8S gene, and ITS2), and the 5′ end of the 28S rRNA gene. Genomic DNA was isolated from the maxillary tissue using a DNeasy blood and tissue kit (Qiagen, Ventura, CA, USA) according to the manufacturer’s instructions. For polymerase chain reaction (PCR), a set of primers previously reported for fungal species (ITS1–5′-TCCGTAGGTGAACCTGCGG-3′ and ITS4–5′-TCCTCCCGCTTATTGATATGC-3′) was used [8, 9]. The amplicon was purified, and the nucleotide sequence was determined in both directions with Taq FS dye-terminator cycle-sequencing fluorescence-based sequencing and analysed on an Applied Biosystems 3730 xl DNA sequencing system. A PCR product of approximately 730 bp was amplified from the maxillary sample. The PCR product was purified and sequenced in both directions using the same primers. A consensus sequence homology search was performed in the GenBank database (nucleotide BLAST); the obtained sequence showed 100% homology with the *Apophysomyces ossiformis* UTHSC 04–838 strain, 99% homology with the *A. ossiformis* UTHSC 07–204 strain, and 94% homology with the *A. thailandensis* SDBR-CMUS219 and *A. trapeziformis* DUM101.13 strains.

Afterward, the patient was kept in the intensive care unit for 7 days and then transferred for continued hospitalization with an antibiotic regimen of piperacillin-tazobactam IV 4.5 g/6 h and liposomal amphotericin B (10 mg/kg/day). On day 15, the patient requested to return to the General Hospital of Tlaxcala to continue treatment. The patient died 9 days after admittance due to complications associated with his poorly controlled diabetes. Pneumonia caused by *Klebsiella pneumoniae* was present.

## Discussion and conclusion

The global guideline for the diagnosis and management of mucormycosis was recently published by the European confederation of medical mycology. This initiative aims to generate actions that reduce mortality in patients through a rapid and specific diagnosis and therapeutic intervention, complemented with assistance of team surgery, radiology, and mycology laboratory [10].

Regarding the aetiology of mucormycosis, *Rhizopus* and *Mucor* spp. are found in 80% of cases; however, in recent years, *Apophysomyces* spp. have emerged as pathogens in both immunocompetent and immunocompromised individuals [5, 6, 11]. Although *A. elegans* was historically considered the only pathogenic species, other species (*A. mexicanus*, *A*. *ossiformis*, *A*. *trapeziformis* and *A. variabilis*) have been recently reported as infection-causing agents [5–7]. The only case of infection by *A. ossiformis* was reported in an immunocompetent patient with humeral osteomyelitis [7]. In this work we report a rhino-orbital mucormycosis due to *A. ossiformis* in a patient with diabetes mellitus.

The incidence of infections due to *Apophysomyces* spp. has been frequently underestimated because the isolation and identification of *Apophysomyces* spp. is not easy, as it requires the use of culture media deficient in nutrients (Czapek-Dox agar), high incubation temperatures for growth (37 °C–42 °C), and prolonged incubation times (7–10 days); additionally, their morphological characteristics often overlap with those of other species, hindering identification at the species level and thus leading to the need for molecular techniques [11, 12].

In our clinical case, the fungal isolation was unsuccessful because it was performed in Sabouraud dextrose agar, a culture medium that is not appropriate for the growth of *Apophysomyces* spp., as well as for the harsh processing of tissue. However, the visualization of hyphae characteristic of Mucorales allowed rapid action, such as surgical intervention in the affected area, the beginning of treatment and molecular identification.

From a clinical point of view, *Apophysomyces* can cause necrotizing fasciitis and renal and rhino-orbital-cerebral infections, and the treatment of choice is amphotericin B. However, research in a murine model showed the efficacy of posaconazole for the treatment of disseminated infections by *A*. *variabilis*. Further clinical studies are needed to determine the potential use of this antifungal agent in human infections [11, 13]. Amphotericin B is the first-line antifungal monotherapy recommended by the global mucormycosis guide. There is no effective combination therapy so far; there are limited data about combinations of polyenes with azoles or polyenes with echinocandins [10].

Recently, the use of isavuconazole (a new antifungal drug) has been reported in cases of cutaneous and disseminated mucormycosis, rhino-orbital mucormycosis, and different *Rhizopus* species, as well as in *A. elegans*. The potent activity against these species shows that isavuconazole can be used as a primary or salvage treatment for mucormycosis [14–17].

The different Mucoral species are morphologically similar, which makes difficult the identification of species using mycological culture and morphological description. Because of this, the use of molecular techniques for identification is highly recommended and preferred. For molecular identification, the use of fresh material is better than formalin-fixed paraffin-embedded tissue because the formalin damages DNA. Among the most used molecular markers are the ITS, 18S, 28 s rRNA and cytochrome B. The global mucormycosis guide, the Clinical and Laboratory Standards Institute (CLSI) and the ISHAM Working Group on Fungal Molecular Identification coincide in recommending amplification and sequencing of the ITS region, as the most precise and accurate method for species identification [18–20].

Another alternative method for rapid identification is the MALDI-TOF mass spectrometry, especially when an in-house database is used and it is compared to commercial databases. MALDI-TOF is a promising technique that still requires validation, as well as a robust commercial database availability [21–23].

Notably, in this case, the aetiologic agent was identified as *A. ossiformis*, which constitutes, to the best of our knowledge, the first case rhino-orbital mucormycosis and the second case worldwide report of infection by this fungus. This case reveals the need to identify the fungus causing mucormycosis with molecular methods to identify adequate treatment therapies for patients, as it has been reported that *A. variabilis* is more susceptible to posaconazole than to amphotericin B, the treatment of choice for mucormycosis. However, the responses of the other species in this genus to antifungal agents are currently unknown, and it is not known whether virulence differs among them.

## Data Availability

The datasets generated and/or analysed during the current study are available from the corresponding author on reasonable request.

## Abbreviations

CT: *Computed tomography*
ITS: Internal transcribed spacer IV: intravenous
KOH: Potassium hydroxide
